# Recent Advances in Understanding the Human Fungal Pathogen Hypoxia Response in Disease Progression

**DOI:** 10.1146/annurev-micro-032521-021745

**Published:** 2023-09-15

**Authors:** Charles Puerner, Sandeep Vellanki, Julianne L. Strauch, Robert A. Cramer

**Affiliations:** 1 Department of Microbiology and Immunology, Geisel School of Medicine at Dartmouth, Hanover, New Hampshire, USA; 2 Department of Biology, Dartmouth College, Hanover, New Hampshire, USA

**Keywords:** hypoxia, oxygen, fungal virulence, disease progression, antifungal drugs

## Abstract

Fungal-mediated disease progression and antifungal drug efficacy are significantly impacted by the dynamic infection microenvironment. At the site of infection, oxygen often becomes limiting and induces a hypoxia response in both the fungal pathogen and host cells. The fungal hypoxia response impacts several important aspects of fungal biology that contribute to pathogenesis, virulence, antifungal drug susceptibility, and ultimately infection outcomes. In this review, we summarize recent advances in understanding the molecular mechanisms of the hypoxia response in the most common human fungal pathogens, discuss potential therapeutic opportunities, and highlight important areas for future research.

## INTRODUCTION

Change is a constant in life, and the relationships between humanity and fungi are no exception. References to human disease caused by fungi were limited prior to the mid-twentieth century but are now a daily occurrence in hospitals across the world. Advances in medical therapies for numerous acute and chronic diseases have opened up new opportunities for fungi to take advantage of humans as hosts. Moreover, emerging infections by other organisms such as viruses have created new opportunities for human fungal infections. As our global climate warms, the thermal barrier to human fungal infections is also expected to shift, resulting in more fungal-mediated human disease (17). The changes in the incidence and scope of fungal-mediated human diseases are rather remarkable, and their importance is emphasized by the persistence of suboptimal fungal infection treatment outcomes. All these factors led the World Health Organization to identify several fungi as critical and high-impact human pathogens in need of further study and targeted therapies (124).

In the face of the increasing incidence of fungal-mediated human disease, contemporary anti-fungal therapies have arguably not kept pace (106). We still rely on drugs from only four approved classes to treat most infections: the azoles, polyenes, pyrimidines, and echinocandins. Intrinsic resistance to these drugs is rapidly increasing, and even when a fungal isolate has antifungal drug susceptibility in standardized in vitro assays, positive infection outcomes do not always follow treatment. Many mechanisms potentially drive poor infection treatment outcomes with a drug susceptible isolate(s), but the dynamic nature of the infection microenvironment is likely a significant, and still ill-defined, factor (95).

**Figure 1:**
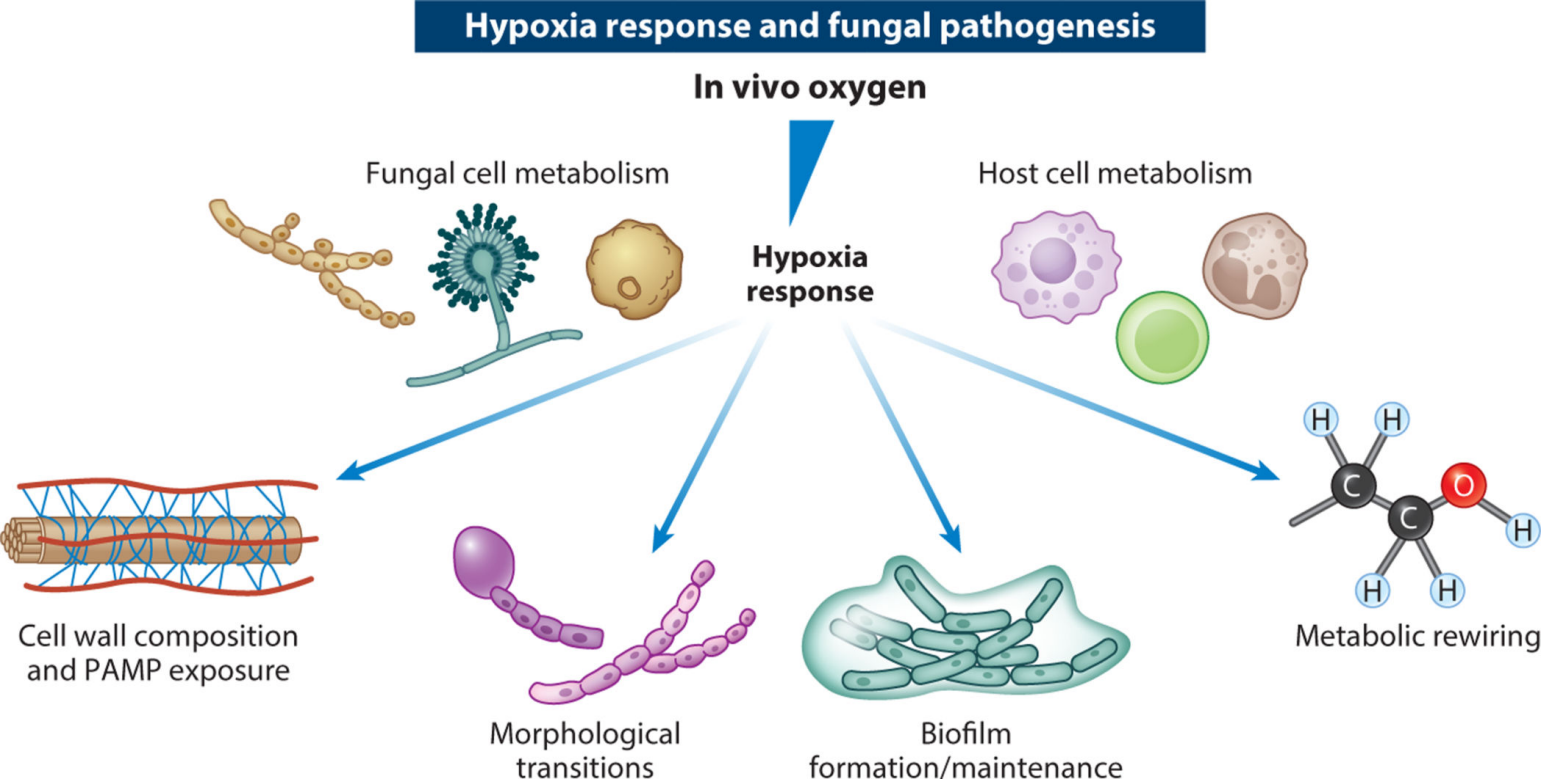
Impacts of fungal hypoxia response on disease progression factors. At the site of infection, oxygen is consumed by both fungal and host cells. Structural damage to the vasculature also often occurs and impairs oxygen delivery. As oxygen becomes limiting, the fungal hypoxia response ensues and impacts multiple disease progression factors—including the fungal cell wall, morphological transitions, biofilm formation and maintenance, and metabolic rewiring—that also may affect antifungal drug susceptibility. Adapted from images created with BioRender.com. Abbreviation: PAMP, pathogen-associated molecular pattern.

Changes in the nature of the infection microenvironment alter host and fungal cell physiology in unclear ways that alter drug efficacy. One key component essential to the viability and physiology of both fungal and host cells is oxygen (43, 55). Atmospheric oxygen levels have varied widely throughout the evolution of life on Earth, as they also vary in the infection microenvironment (2, 37). Oxygen is the most used substrate in metabolic reactions and is critical for the biosynthesis of sterols, heme, and NAD^+^ (105). It is also a thermodynamically efficient terminal electron acceptor used to generate chemical energy. Consequently, fungi have evolved sophisticated physiology that relies largely on oxygen. How fungi deal with the dynamic changes in oxygen availability found in various ecosystems fungi inhabit, including the human body, remains an active area of investigation. pO_2_ (partial pressure of O_2_) levels vary throughout the human body and are substantially lower than pO_2_ levels utilized in most laboratory-based experiments of fungal virulence mechanisms and antifungal drug susceptibility (6). Most healthy tissues have molecular oxygen (O_2_) levels between approximately 2.5% and 9%, but most in vitro fungal pathogenesis studies are conducted in standard atmospheric conditions (21% O_2_, or pO_2_ ∼160 mmHg). While exact O_2_ levels in a given environment are affected by atmospheric pressure and CO_2_ levels, 20–21% oxygen levels are generally referred to as normoxia in the literature. Following tissue damage, including damage from infection, oxygen levels can drop considerably lower than 5%, making oxygen a limiting factor in host and microbial cell physiology. Oxygen levels of less than 5% are often described in the literature as hypoxia. When environmental oxygen levels do not meet fungal or host cell demands, a hypoxia response is initiated. How the fungal hypoxia response impacts fungal pathogenesis, virulence, and drug susceptibility is the focus of this review, which builds on previous discussions of this important topic (2, 24, 43, 55). Figure 1 summarizes some of the key impacts of the hypoxia response on fungal-host interactions.

As introduced above, terminology in the literature discussing oxygen levels and related host-fungal interaction physiology can be confusing and imprecise. It is difficult at times to measure the absolute levels of oxygen fungi are experiencing in controlled studies. Some of the variability in findings reported in the literature may in fact be due to differences in the use of the term hypoxia. The hypoxia response of an organism is determined not by an absolute level of oxygen but rather a physiological response to oxygen cellular demand not being met by oxygen availability in the local environment. A cell’s demand for oxygen, and its utilization, will be impacted by other microenvironmental variables such as available macro- and micronutrients, pH, partial pressure, temperature, and CO_2_ levels, among others. Consequently, it is important to realize the distinction between hypoxia viewed from a physiological response perspective and hypoxia denoting low levels of environmental oxygen. The importance of this distinction is highlighted by fungal biofilms. Typically formed in normoxic conditions in laboratory experiments, fungal cells within the developing and mature biofilm experience a low-oxygen, often referred to as hypoxic, environment likely due to the rapid consumption of oxygen by exterior cells (49, 74, 75). When possible, the absolute level of oxygen present in the environment during a specific experiment should be explicitly stated. The term microoxia is often used in reports of laboratory-based microbiology studies where conditions of oxygen deficiency are induced to investigate the associated molecular mechanisms. Whether microoxic conditions induce a hypoxia response in a given organism, particularly fungi, depends on multiple environmental variables. Studies with pathogenic fungi in anoxic conditions, completely lacking molecular oxygen, are limited but an important area for further research. The relative lack of anoxic studies is related to the fact that the most common human fungal pathogens do not grow via anaerobic processes unless specific oxygen-dependent nutrients are added to the growth medium. More research in fungi is needed to define specific hypoxia response biomarkers to enable detection of fungi experiencing physiologic oxygen deprivation.

As fungal infections initiate in different host tissues, it is likely that fungal cells experience oxygen deprivation in vivo at least in part from fungal metabolic activity. Both *Aspergillus fumigatus*, a mold, and *Cryptococcus neoformans*, a yeast, are considered obligate aerobes and rely on mitochondrial respiration for growth in environments with dynamic oxygen levels. Conversely, the human commensal *Candida albicans* is a facultative anaerobe capable of utilizing mitochondrial respiration or fermentation pathways for growth in the human gastrointestinal tract and other sites of infection. At the site of infection, neutrophils consume substantial quantities of oxygen to drive NADPH oxidase activity for antimicrobial defense. Direct evidence for depletion of oxygen levels at the site of a fungal infection has been observed in murine models of fungal disease. In immunologically distinct murine models of invasive pulmonary aspergillosis, a nitroimidazole derivative (hypoxyprobe-1) was utilized to detect regions of the lung with oxygen levels at or below 1.5% ambient (54). A similar reagent detecting low-oxygen tissue was utilized to observe significant hypoxic regions and granuloma-associated immune cells in a murine model of histoplasmosis (38). In a murine model of systemic candidiasis, regions of low oxygen were detected in the kidney utilizing a carbonic anhydrase–based method (83). However, the dynamics of oxygen availability to fungal pathogens and host cells at the site of infection remains to be fully explored and is an important topic for investigation because understanding mechanisms driving oxygen depletion during infection is expected to yield new therapeutic opportunities. Moreover, modeling the in vivo oxygen dynamics faced by the fungal pathogen may allow refinement of in vitro culture conditions to more closely replicate the host environment. It is worth considering that in many in vitro experiments, fungi are grown under normoxia conditions (standard atmosphere) that are not found in the human body. Even in the airways of the lung, the tissue with the highest oxygen availability, oxygen levels are around 14.5%.

Support for the conclusion that reduced oxygen levels in vivo affect the outcome of fungal-host interactions is multifaceted. Organisms with mutations in genes (Table 1) that are required for fitness under in vitro conditions with oxygen levels similar to those observed in vivo are often severely attenuated in virulence in murine models. Many of these genes are thus important regulators or components of the fungal hypoxia response. Loss of key regulators of the mammalian hypoxia response also confers increased susceptibility to fungal infections in murine and cell culture models (46, 114). Intriguingly, modulation of in vivo oxygen levels utilizing hyperbaric oxygen alters fungal disease progression in murine models and has had some success in humans, as evidenced in clinical case reports (35). While much has been learned in recent years on how fungi alter their virulence in response to dynamic oxygen levels, it is clear we still have a long way to go in understanding how oxygen impacts fungal virulence mechanisms and antifungal drug susceptibility. An emerging theme is the importance of oxygen in metabolic rewiring that alters host immune system recognition and clearance of fungi. Moreover, as the role of fungi in the microbiota becomes more apparent, oxygen is at the interface of fungal-microbe interactions in the gastrointestinal tract through mechanisms that remain to be fully defined. It will be interesting to see whether fungal genes required for the virulence-associated hypoxia response are also involved in fungal commensalism. Below we review and discuss some emerging themes around the human pathogenic fungi hypoxia response.

## OXYGEN AND FUNGAL DISEASE PROGRESSION: FUNGAL MORPHOGENESIS AND SENSING

**Figure 2:**
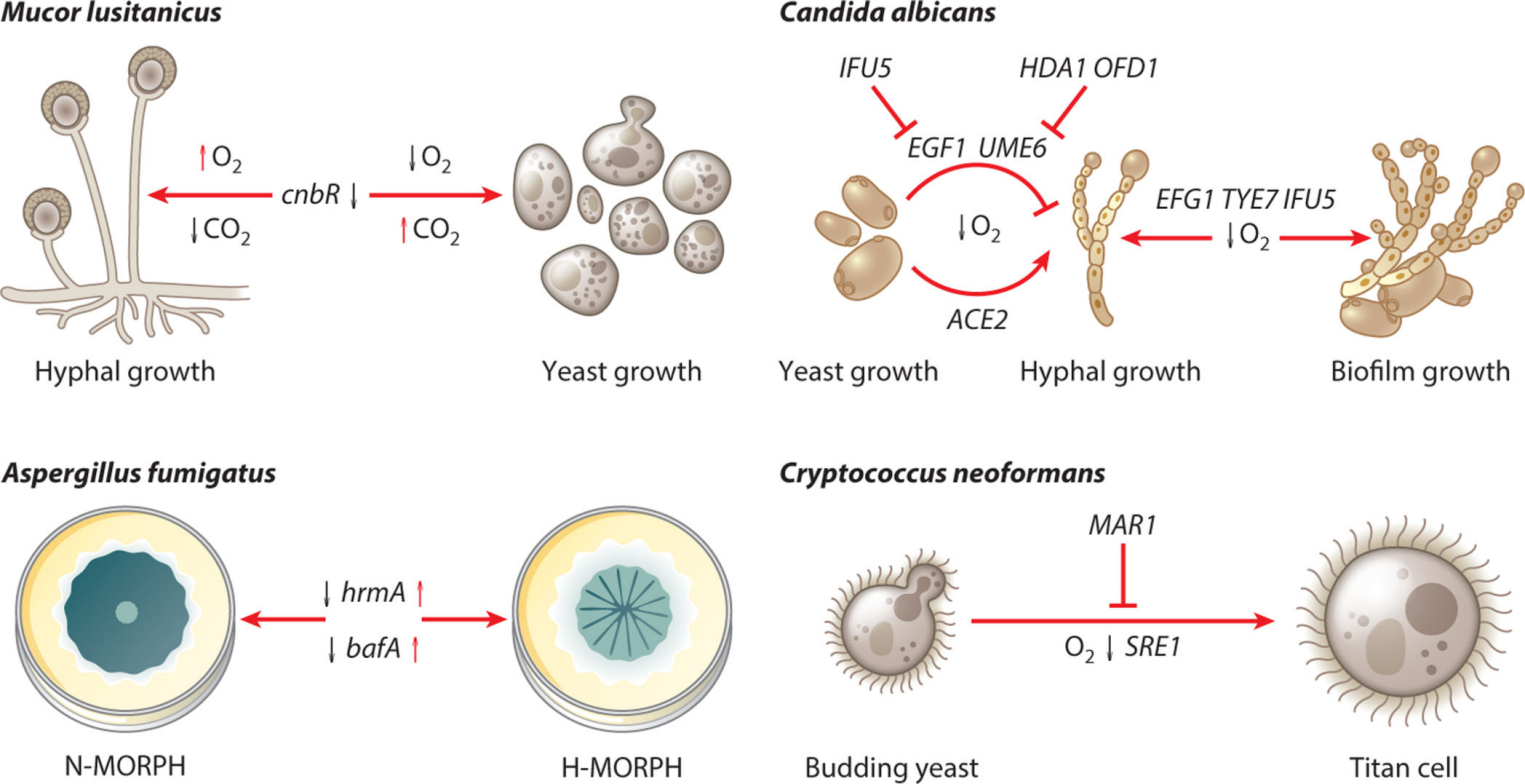
Hypoxia response–induced morphological changes of pathogenic fungi. Genes and atmospheric conditions are indicated that have roles in morphological changes for the fungal pathogens *Mucor lusitanicus*, *Candida albicans*, *Aspergillus fumigatus*, and *Cryptococcus neoformans.* These morphological changes occur at least in part due to hypoxic conditions in the host infection environment. Morphological changes for the dimorphic fungi *M. lusitanicus* and *C. albicans* are characterized by the transition between yeast and hyphal forms. The *C. neoformans* hypoxia response plays a role in the transition to titan cell formation. Lastly, the morphological switch from N-MORPH to H-MORPH biofilms in *A. fumigatus* is driven by repeated exposure to low oxygen. Together these responses result in more virulent morphotypes; therefore, research into the biology and mechanisms of these changes is expected to lead to new therapeutic opportunities. Adapted from images created with BioRender.com.

Fungi display a remarkable array of morphological transitions during their life cycles in nature and during human infections. These transitions have long been studied in the context of their impact on disease progression (Figure 2). The ability to switch between yeast and filamentous growth, for example, is important to the success of *C. albicans* as a human pathogen, as mutant strains unable to undergo this switch are avirulent in a systemic candidiasis murine model (82). An important step for the initiation of infection by *C. albicans* in the intestine is the transition from the yeast form to the filamentous form. During this transition, hyphal-specific genes are turned on, leading to increased adherence and excretion of lytic enzymes (e.g., Ece1) among others (1, 52, 123). Several environmental signals have been identified that lead to this transition, among which is low oxygen (for a review see 19). The transcription factors Efg1 and Ace2 have been implicated as important regulators of filamentation in *C. albicans* (70, 71, 93, 117, 118). The roles of Efg1 and Ace2 in regulation of filamentation in the context of host-relevant atmospheric environments have been described (12, 34, 112). During low-oxygen conditions, Efg1 represses filamentation signals, whereas Ace2 promotes filamentation (34, 112). This opposition has been hypothesized to be important for the success of *C. albicans* as a commensal, but further research is needed. An additional level of regulation of Efg1 activity is accomplished by the WW domain–containing protein Ifu5. Transcription of *EFG1* in oxygen-replete conditions is repressed through the WW domain–mediated binding of Efg1 by Ifu5. The transition to a low-oxygen environment relieves this regulation, allowing for the transcription of *EFG1* and the downstream effects of Efg1 in oxygen-limited environments (104). An in silico study recently reported the possibility of utilizing naturally occurring polyphenols to regulate Efg1 activity as a possible future treatment option (90). How *C. albicans* senses dynamic oxygen levels to initiate these responses remains an open question.

Recently a genetic screen of *C. albicans* mutant strains from the thematic transcriptional regulator libraries was conducted to identify mutant strains defective in growing on alternative carbon sources (sucrose and glycerol) in a 5% O_2_ environment (16). This screen identified two mutants with a significant growth defect on alternative carbon sources in low oxygen, *tye7*Δ and *snf5*Δ. The gene *TYE7* was previously implicated in the *C. albicans* hypoxia response through its regulation of genes involved in glycolysis (4, 10). However, the involvement of the switch/sucrose nonfermentable (SWI/SNF) chromatin-remodeling complex component Snf5 in the low-oxygen response is a new finding. The role of Snf5 in low-oxygen conditions appears to be through interaction with the adenylate cyclase Cyr1 to regulate metabolic flexibility through the Ras-cAMP–protein kinase A (PKA) pathway that is further discussed below (16). Tye7 was also recently identified in another genetic screen as a negative regulator of hyphal growth in low-oxygen conditions along with the transcription factor Ahr1 (61). These transcription factors have been previously described as being involved in carbon metabolism (Tye7) and expression of adhesins (4, 5, 111). The null mutants *tye7*Δ and *ahr1*Δ were hyperfilamentous, specifically in the presence of 1% O_2_. Through epistatic analysis it was discovered that Tye7 and Ahr1 act independently in two separate pathways, the Ras1/Cyr1 cAMP pathway and the Efg1 pathway, respectively (61). The *ahr1*Δ mutant had a transcriptional profile indicating iron starvation, thus possibly the hyperfilamentation phenotype is for environment exploration and nutrient uptake.

As mentioned, how fungi sense changing oxygen levels to adjust morphology and metabolic responses remains ill-defined. Indirect sensing of oxygen-dependent metabolites such as sterols, heme, and NAD+ has been studied. Direct oxygen sensors have been identified in other organisms, with the seminal example being prolyl 4-hydroxylase (PHD)-mediated regulation of the mammalian transcription factor hypoxia-inducible factor 1 (HIF-1) (13, 42, 68, 69, 89, 121). In dimorphic fungi, the hyphal-specific transcription factor Ume6 is also important for the initiation and maintenance of hyphal growth and regulated through a PHD-mediated mechanism (85). Ume6 is dually regulated by chromatin remodeling via Hda1 and protein stability by the prolyl 4-hydroxylase-like 2-oxoglutarate-Fe(II) dioxygenase, Ofd1 (85, 86). More recently it has been discovered that Ume6 protein stability is regulated by both low oxygen and CO_2_ levels (84). Both low oxygen and physiological CO_2_ promote the stability of Ume6, allowing filamentation to proceed (84). PHD-mediated regulation of the sterol regulatory element binding protein (SREBP)-mediated low-oxygen response through Ofd1 homologs is well studied in fission yeast, but its overall role in fungal pathogenesis remains ill-defined despite Ofd1 homologs being present in many human-pathogenic fungi (25, 66, 79).

Other proteins that bind or utilize oxygen are candidate oxygen sensors. In *A. fumigatus*, the gene *fglA* encodes a protein termed a fungoglobin with a globin-like structural domain at the protein’s N terminus that was confirmed to bind a heme cofactor and oxygen (62). Loss of *fglA* impaired fungal growth in environments with oxygen levels below 0.5% and induced a fluffy, aerial hyphal colony biofilm morphology (62). An *fglA* homolog was also characterized in *Paracoccidioides brasiliensis* and observed to be required for optimum growth in low-oxygen conditions (94). In vivo, *fglA* transcripts were elevated in yeast isolated from murine lungs 12 h after *P. brasiliensis* infection (94). However, loss of *fglA* did not affect host survival in a murine model of invasive pulmonary aspergillosis (62). It is intriguing to speculate that *fglA* is critical in extreme oxygen-limited environments and/or during long-term chronic infections that are difficult to model in mice. Its role as an oxygen sensor and fungal virulence factor remains to be fully explored, and the extent to which other human pathogenic fungi rely on a fungoglobin is ill defined.

Redox-sensitive molecules may also serve as oxygen sensors in fungi. Proteomic analysis of the *A. fumigatus* response to low-oxygen conditions revealed oxidized proteins including those involved in protein folding, redox balance reactions, and the mitochondrial respiratory chain (113). The mitochondrial respiratory complex IV assembly protein Coa6 was oxidized in low-oxygen conditions, and its loss completely eliminated growth in a hypoxic environment (113). These observations built on earlier work in *C. neoformans* and *A. fumigatus* clearly indicating an important role for the mitochondria and electron transport chain in the fungal pathogen hypoxia response (53, 67, 76, 77). The role of hypoxic reactive oxygen species (ROS) signaling in the fungal hypoxia response is an exciting area for further exploration in pathogenic fungi.

Disease progression and morphogenesis of another human fungal disease, mucormycosis, caused by fungi of order Mucorales, are also impacted by oxygen levels (Figure 2). These fungi are ubiquitous and enter the host via inhalation of spores or skin cuts/wounds (51). Pulmonary mucormycosis is characterized by hyphal invasion of tissues and blood vessels, which results in excessive necrosis, all of which contribute to the formation of low-oxygen microenvironments at the site of infection. For example, disease progression during the outbreak of mucormycosis during the COVID-19 pandemic, particularly in India, has been hypothesized to be partly due to the growth of mucormycosis-causing fungi in the hypoxic conditions generated by the viral infection (36, 109). Additionally, adjunctive hyperbaric oxygen treatment, which may increase oxygen levels at the site of infection, has been reported to improve clinical outcomes in some patients with rhinocerebral mucormycosis, further highlighting the role of oxygen in the pathogenesis of mucormycosis (47).

In the order Mucorales, only *Mucor* species exhibit dimorphism (yeast and hyphal morphology). Much remains to be learned about how dynamic oxygen levels impact the biology of these important human pathogens. Oxygen is a positive regulator of hyphal development in *Mucor*, as growth under normoxic conditions, which promotes oxidative metabolism, results in hyphal development and biofilm formation. In contrast, growth under low-oxygen and high–carbon dioxide conditions promotes fermentative metabolism resulting in the formation of yeast-like cells (101) (Figure 2). Inhibition of components of the electron transport chain and oxidative phosphorylation by chemicals results in yeast growth even in normoxic conditions, further suggesting oxidative metabolism is required for hyphal development (reviewed in 98). Carbon dioxide and oxygen likely promote hyphal development in *Mucor* species by suppressing cAMP-dependent PKA activity. PKA activity is lower under normoxic conditions but increases significantly under low-oxygen conditions to promote yeast growth. The increase in PKA activity also suppressed Efg1 activity (not to be confused with *C. albicans* Efg1, the transcription factor). However, the precise role of Efg1 in *Mucor* hyphal development and disease progression is yet to be determined (92).

A second major positive regulator of hyphal development in *Mucor* species is calcineurin, a calcium- and calmodulin-dependent phosphatase. A loss-of-function mutation in the regulatory subunit of calcineurin (cnbR) in *Mucor lusitanicus* (also known as *M. circinelloides* f. *lusitanicus* and previously referred to as *Mucor circinelloides*) results in a yeast morphology correlated with an increase in PKA activity in a cAMP-independent manner (80). However, prolonged growth of the *cnbR* mutant results in the emergence of hyphal sectors from yeast-locked colonies. Whole-genome sequencing of these hyphal sectors revealed loss-of-function mutations in a novel amino acid permease gene (*bycA*) (122). Deletion of both *cnbR* and *bycA* results in hyphal growth. Together the data suggest that active calcineurin prevents PKA activation by suppressing *bycA* expression and promoting hyphal growth. It is unlikely that neither calcineurin nor BycA has a role in *Mucor* growth in anaerobic conditions, as the *cnbR bycA* double mutant exhibits yeast growth. However, identifying direct calcineurin targets in low-oxygen conditions is expected to provide more mechanistic insights into morphogenesis, hyphal development, and disease progression mechanisms.

The protein kinase regulatory subunit (*pkr1*) and G-protein beta subunit (*gpb1*) also promote hyphal development in low-oxygen conditions in *M. lusitanicus*, as deletion of these genes accelerates yeast growth and switch to fermentative metabolism under low-oxygen conditions (120). Deletion of *gpb1* results in reduced virulence in murine models, though the extent of oxygen deprivation in murine models of mucormycosis remains to be defined.

While *Mucor* yeast morphology is rarely reported in mucormycosis patients, case studies have reported yeast-like cells in mucormycosis patients’ blood and urine samples (3, 26). Thus, the dynamics of the infection microenvironment may impact low-oxygen signaling and *Mucor* morphogenesis in complex ways we do not fully understand. For example, calcineurin may still be active under hypoxic conditions in vivo, promoting hyphal growth and suppressing the yeast phase. Intriguingly, it has been reported that inhibition of lipid biosynthesis (by cerulenin and cycloleucine) blocks *Mucor* yeast-to-hyphae transition; however, this can be overcome by supplementation with Tween 80 (reviewed in 98). It is possible that *Mucor* takes up fatty acids from the serum during an active infection, which promotes hyphal growth. This hypothesis is supported by the observation that low oxygen increases the expression of genes involved in lipid metabolism and endocytosis in *Mucor irregularis* (127). Importantly, in addition to low oxygen, high carbon dioxide levels are required for yeast growth. While it is possible that carbon dioxide levels at the site of infection may not be high enough to promote yeast growth, sites of tissue oxygen deprivation are also typically high in carbon dioxide levels due to cellular metabolism. What seems clear is that dynamic environmental conditions in vivo, yet to be fully defined, can impact oxygen signaling mechanisms and phenotypes in pathogenic fungi.

An important recent morphological discovery for *C. neoformans* was titan cell formation in vivo (97, 128). *C. neoformans* titan cells are characterized as polyploid, enlarged cells greater than 15 μm and upwards of 100 μm in diameter. Additionally, titan cells have a thicker cell wall and a denser capsule (97, 128). Importantly these morphotypes are incapable of being phagocytosed and can produce normal-sized prodigy by budding, which is likely important for dissemination and disease progression (28, 97, 128). The presence of titan cells has also been shown to reduce phagocytosis of normal sized-prodigy, further evidence that titan cells promote virulence (96). Several host-relevant conditions for in vitro titan cell formation have been described, among which is exposure to low-oxygen conditions (63) (Figure 2). Hommel et al. (63) also showed that this morphogenesis is partially dependent on the hypoxia response SREBP transcriptional regulator Sre1. Recently the gene *MAR1*, which regulates cell wall composition and immune evasion, was shown to contribute to hypoxia stress resistance and titan cell formation, highlighting hypoxia stress as an inducer of this altered morphology (44, 119). Interestingly the *mar1*Δ mutant can form granuloma-like structures in the murine lung that are expected to be hypoxic (119).

## FUNGAL BIOFILMS—LOW-OXYGEN NICHE SPECIALIZATION AND ANTIFUNGAL DRUG RESISTANCE

Formation of a multicellular biofilm structure results in increased resistance to treatments and increased success by the pathogen. *C. albicans* biofilms form in several host niches and are particularly problematic on medical devices. Due to their complex structure, biofilms are difficult to treat and clear from the host. The transcriptional activator of glycolytic genes discussed above, Tye7, is also implicated in biofilm formation (4, 10). *TYE7* is an SREBP homolog most similar to *srbB* in *A. fumigatus* and *sre2* in *C. neoformans*. Bonhomme et al. (10) reported that null *tye7*Δ mutants display an increase in filamentous growth and do not form fully mature biofilms. This work links biofilm formation to metabolism, specifically glycolysis in the context of Tye7. Given the finding that Ahr1 works in opposition to Tye7 through Efg1 regulation, it is worth noting that positive autoregulation of Efg1 and through Ifu5 is important for the formation of biofilms under hypoxic conditions (61, 104, 116). Moreover, in *C. albicans*, SREBPs have been repurposed for gene activation of hypoxia-responsive genes but do not regulate ergosterol biosynthesis, as this is controlled by the Upc2 transcription factor in this organism (88, 131). The difference in low oxygen sterol biosynthesis regulation illustrated by the Upc2/SREBP rewiring across fungal pathogens highlights the need to study the hypoxia response in each respective fungal pathogen. The connection between oxygen levels, biofilm formation, and metabolism is important when considering possible novel antifungal drug targets and treatments. Targeting an aspect of metabolism required for fungal hypoxia adaptation is expected to decrease or eliminate the ability to form fungal biofilms.

Recent work has combined transcriptomic, metabolomic, and secreted proteomic approaches to address how amino acid acquisition and metabolism impact biofilm development (11). This study investigated several conditions and found that in low oxygen, *C. albicans* biofilms have an initial period of adaptation during which dynamic metabolic rewiring occurs and leads to similar profiles as observed in aging biofilms (11). These data further support the observations that a mature *C. albicans* biofilm experiences hypoxia and undergoes metabolic rewiring. This is likely the case for other fungi where metabolic rewiring occurs during biofilm development driven by oxygen and nutrient limitation, an area that warrants further investigation. For example, genes involved in biofilm formation in other *Candida* species such as *Candida parapsilosis* are similar to those involved in the hypoxia response (e.g., glycolysis, fatty acid metabolism, and ergosterol biosynthesis), further supporting the tight connection between hypoxia adaptation and biofilm formation and function (108, 110, 112).

Additionally, low oxygen has been shown to increase biofilm formation of *Candida glabrata* in the context of the host-relevant minimal media RPMI 1640 but decrease it in the context of the rich media YNB (59). *C. glabrata* null mutants that were known to be deficient in biofilm formation in normoxic conditions had an increase in biofilm formation in hypoxic conditions (57). Importantly, low oxygen also drives resistance to amphotericin B in RPMI 1640 but not YNB (59). This effect is like that observed in filamentous fungi where low-oxygen environments are a driver of antifungal drug resistance in biofilms grown in glucose minimal media (74). These observations highlight an important open question on how nutrient limitation and low oxygen in the biofilm leads to increased antifungal drug resistance. Organisms with null mutations in genes involved in cell wall biosynthesis, the DNA checkpoint pathway, or ergosterol biosynthesis or in genes for transporters or transcription factors were found to have reduced biofilm formation when grown in the presence of fluconazole (57). Additionally, the constitutive expression of *CAGL0M02233g*, a homolog of the *Saccharomyces cerevisiae RAD53* DNA checkpoint effector, led to decreased biofilm formation and increased fluconazole susceptibility (58). This provides insight into possible drug targets (genes and pathways) that could be leveraged to prevent the formation of fungal biofilms. Further research is needed to fully address the mechanisms by which hypoxic conditions and nutrient deprivation drive antifungal drug resistance in mature biofilms.

An emerging area of fungal biofilm research is its role in polymicrobial interactions and infections. Biofilms at infection sites are often polymicrobial, coinhabited by multiple species of fungi or bacteria. A crucial area of future research will investigate how polymicrobial communities are able to work together to overcome the stresses experienced during infection and biofilm formation. A recent transcriptomic-based study of *C. albicans* cocultured with the gram-negative bacterium *Pseudomonas aeruginosa* investigated the transcriptional response of this coculture in static biofilms (48). Fourie and coworkers (48) reported that the transcriptional changes that occur in *C. albicans* during coculture with *P. aeruginosa* were like those of the transcriptional response to hypoxia. Given this result, it seems that *P. aeruginosa* coculture may induce a hypoxic state in *C. albicans* biofilm cells and have implications for pathogenesis and important phenotypes such as antifungal drug resistance that remain to be fully explored.

Connections between fungal biofilms and hypoxia are also found in the filamentous fungal pathogens. *A. fumigatus* disease initiation and progression involves conidial germination, hyphal expansion, and biofilm formation, resulting in tissue and blood vessel invasion and inflammation, which leads to hypoxic infection microenvironments. It is possible that reducing hypoxic zones at the infection site might improve disease outcomes. In support of this hypothesis, Ben-Ami et al. (9) observed that when *A. fumigatus*–infected mice were treated with proangiogenic growth factors FGF-2 and VEGF alone or combined with antifungal drugs, host survival increased. These data indicate that hypoxic infection microenvironments promote *A. fumigatus* disease progression.

In a murine model of invasive pulmonary aspergillosis, strains of *A. fumigatus* that generate more biomass in hypoxic conditions in vitro correlate with increased virulence (72). Moreover, *A. fumigatus* strains with null mutations in genes encoding transcription factors critical for fungal growth in hypoxic environments such as *srbA*, *srbB*, *atrR*, and *creA* all display significant attenuations in virulence (8, 23, 60, 125). Intriguingly, fungi with null mutations of the hypoxia response transcriptional regulators *srbA*, *srbB*, and *creA* fail to form fully mature biofilms, strongly suggesting the *A. fumigatus* hypoxia response is critical for biofilm maturation, as observed in *Candida* species (8, 23, 60, 74, 125). Recently, AtrR and SrbA were observed to coregulate a significant number of genes associated with azole antifungal susceptibility and the fungal hypoxia response, such as *erg3B*, *erg24A*, *erg25A*, *cyp51A*, *aoxA*, and *mdr1*, among others (100). More research is needed to fully define the transcriptional regulatory mechanisms underlying adaptation to low-oxygen environments in *A. fumigatus* biofilms. Whether these hypoxia response transcriptional regulators function similarly in other human pathogenic molds is an open question.

As *A. fumigatus* biofilms mature, oxygen gradients are established within the biofilm, perhaps explaining the dependency on known hypoxia transcriptional regulators for biofilm maintenance and maturation (74). While the mechanisms remain to be fully defined, studies in the model filamentous fungus *Aspergillus nidulans* revealed that biofilm maturation involves the depolarization or disassembly of microtubules at the base of the biofilm and changes in endoplasmic reticulum exit sites, Golgi apparatus, and sites of endocytosis that are dependent on SrbA, thereby establishing dormancy (81, 103). It is unknown whether *A. fumigatus* biofilm maturation involves microtubule depolarization and structural changes to subcellular organelles within the hypoxic regions of the biofilm.

Intriguingly, the morphology of *A. fumigatus* colonies and submerged biofilms in hypoxic conditions vary from that of biofilms induced in normoxic conditions and is also strain dependent (Figure 2). In low-oxygen conditions on a glucose minimal medium, *A. fumigatus* reference strain AF293 colony biofilms exhibit increased colony furrowing and vegetative mycelium on solid agar plates, a morphology recently termed H-MORPH (73). H-MORPH contrasts with colony biofilms grown in normoxic conditions that exhibit little colony furrowing and extensive conidiation and are termed N-MORPH (73). The strain EVOL20, which is locked in the H-MORPH morphotype, was generated through serial passaging of the N-MOPRH parent AF293 in low-oxygen conditions (72, 73). H-MORPH strains were subsequently found in a collection of clinical isolates. In a murine model of invasive pulmonary aspergillosis, H-MORPH strains display increased disease progression compared to N-MORPH strains (73). H-MORPH strains generate more biomass in low-oxygen conditions, suggesting they can optimize their metabolism for growth under oxygen-limiting conditions (73). It appears that the furrows formed in an H-MORPH colony provide increased cellular access to oxygen. In a submerged biofilm model, H-MORPH hyphae organize more horizontally than N-MORPH biofilms (75). In *C. albicans*, wrinkled colony biofilm morphology appears to provide increased access to oxygen, highlighting the importance of population-level morphology to oxygen access in human fungal pathogens (91). Many questions remain about the function of H-MORPH in *A. fumigatus* pathobiology and the underlying genetic mechanisms that are responsible for its induction and maintenance. Genome sequencing of the EVOL20 isolate revealed a subtelomeric gene cluster termed hypoxia-associated cluster (HAC) that contained a fungal-specific gene, *hrmA*, necessary to induce H-MORPH formation. Subsequent study revealed that HrmA regulates expression of genes in HAC and that a fungal-specific protein of unknown function, BafA, is sufficient to induce H-MORPH in both *A. fumigatus* and *Aspergillus niger* (73). How BafA drives H-MORPH formation is currently unknown.

## HYPOXIA AND FUNGAL–IMMUNE SYSTEM INTERACTIONS

Given the role of oxygen in fungal metabolism, changing its availability has a significant impact on the polysaccharide-rich fungal cell wall, which is arguably the first fungal structure to interact with the host immune system (Figure 1). A connection between oxygen–cell wall dynamics and microbial virulence has been established in *Mycobacterium tuberculosis* (29). Cunningham & Spread-bury (29) reported that low oxygen results in the formation of thicker bacterial cell walls, which promotes dormancy, survival in granulomas, and latent infections. How differences in cell wall thickness or composition impact hypoxia fitness and virulence in acute and persistent infections of *A. fumigatus* and other human pathogenic fungi is only beginning to be explored. However, the *A. fumigatus* EVOL20 strain discussed above has thinner cell walls compared to the parental strain and more exposed β-glucans on the cell surface (73). These data are consistent with hypoxia also increasing exposure of surface β-glucans in the CEA10 strain of *A. fumigatus* (115). H-MORPH strains appear to induce more inflammation in murine models of invasive aspergillosis, and this observation may be due to the increased β-glucan exposure, though this remains to be definitively determined (73). While the mechanism(s) behind oxygen-mediated cell wall changes remains to be fully defined, low-oxygen conditions dramatically modulate mRNA levels of genes involved in cell wall metabolism and biosynthesis in *A. fumigatus*, with genes such as *fksA*, *agsA*, *chsE*, and *chsF* significantly increased in expression (7, 115). The transcriptional regulator(s) that modulates this hypoxia-specific cell wall response in *A. fumigatus* remains to be identified.

In apparent contrast to *A. fumigatus*, *C. albicans* low-oxygen exposure leads to a decrease in β-glucan exposure and cell wall thinning, resulting in immune system evasion due to lack of PAMP (pathogen-associated molecular pattern) exposure (21, 83, 102). This observation was made in representative strains from four *C. albicans* clades (102). In low-oxygen conditions, the viability of neutrophils is unaffected, but during infection with *C. albicans* neutrophils are unable to mount an attack due to the lack of PAMP exposure from β-glucan masking (83). It is unclear whether the reduced oxygen levels at the site of infection also negatively affect the neutrophil respiratory burst, as they do with some bacterial infections. Moreover, it is interesting to think about the differences between *C. albicans* versus *A. fumigatus* with regard to PAMP masking/unmasking in hypoxic conditions. Given that *C. albicans* is a human commensal living in the low-oxygen gastrointestinal tract, it may have evolved oxygen-mediated immune evasion strategies in contrast to those of the environmental saprophyte *A. fumigatus*, where biomass generation in the low-oxygen environment of a compost pile may lead to increased cell wall biosynthesis needed for survival in a competitive ecological niche. Moreover, hypoxia-induced masking of β-glucans is also observed in other human-associated *Candida* species including *C. tropicalis* and *C. krusei*, but not in *C. parapsilosis* (56, 102).

Mechanisms driving cell wall remodeling in low oxygen have been most extensively studied in *C. albicans*. These studies revealed that mitochondrial function and the cAMP-PKA pathway are required for hypoxic cell wall remodeling. Utilizing genetic approaches, it has been found that *C. albicans* with null mutations in the gene that encodes for adenyl cyclase (*cyr1*Δ) or double null mutations in PKA catalytic subunits (*tpk1*Δ*tpk2*Δ) fails to mask β-glucans under hypoxic conditions. Recall that homologs of these genes also impact the hyphae-to-yeast transition in *Mucor* species. The Goa1 protein and Upc2 transcription factor play roles in mitochondrial homeostasis, and *goa1*Δ and *upc2*Δ mutants fail to mask β-glucans in hypoxic conditions (102). However, this defect can be overcome by adding exogenous cAMP, linking mitochondrial functionality to cAMP-PKA protein signaling. Hypoxia results in an increased production of ROS. However, it remains unknown whether ROS activates the cAMP-PKA pathway resulting in a decrease in surface β-glucans. Interestingly, cell wall changes in *CCR4*-*POP2* null mutants are also due to mitochondrial dysfunction, further highlighting a connection between mitochondrial signaling, and cell wall changes. Although deletion of *CCR4*-*POP2*, which encodes an mRNA deadenylase, decreased the exposure of β-glucans to the same extent as wild type in low oxygen, another study has shown that the overall β-glucan composition in the cell wall of Ccr4-Pop2 mutants is significantly lower than in wild type (30).

A mechanism for trimming β-glucan in *C. albicans* was also discovered through the expression of a secreted exoglucanase, Xog1, which leads to PAMP masking-like effects (21). Transcription of the gene *XOG1* is increased upon exposure to hypoxia or lactate. Lactate is produced by host cells at sites of inflammation as a consequence of hypoxic cell metabolism. The secretion of Xog1 results in β-glucan masking through a proposed mechanism of shaving, or removal of the β-glucan layer (21). Importantly, in *xog1*Δ mutants blocking of exoglucanases results in attenuated virulence and fungal burden, indicating an important role in β-glucan shaving in fungal pathogenesis (21). It has been observed that *C. albicans* cells colonizing body sites with low pH such as the vaginal mucosa likely undergo initial β-glucan masking due to low oxygen exposure to initiate colonization (27). Interestingly in this low-pH environment, β-glucan remasking occurs in a farnesol-dependent mechanism indicative of a cell density effect (27). Perhaps this is a mechanism for *C. albicans* to establish as a commensal in the gut.

Besides low-oxygen-mediated changes in fungal PAMP expression and exposure, survival inside host phagocytes may be mediated in part by the fungal hypoxia response. The intracellular fungal pathogen *Histoplasma capsulatum* requires Srb1, an SREBP homolog, to survive in murine macrophages as a yeast (39). Moreover, reduced expression of Srb1 leads to a striking reduction in murine histoplasmosis (39). These results build on previous observations in *C. neoformans*, *A. fumigatus*, and *C. albicans* that fungal SREBP homologs are critical for the fungal hypoxia response and virulence (4, 18, 22, 125).

## HYPOXIA-MEDIATED FUNGAL COMMENSALISM AND LONG-TERM HOST PERSISTENCE

An important and emerging area of research at the intersection of oxygen and human-associated fungi is mechanisms of commensalism and long-term fungal persistence in a host. As *C. albicans* is one of the best-studied fungal commensals of humans, much of our understanding currently comes from this organism. Yet, new observations are emerging from the study of chronic fungal infections that are increasing our understanding of how fungi persist long-term in a human host. For example, the hypoxia-dependent regulation of white-opaque switching by the transcription factors Wor1 and Efg1 is important for successful gut colonization (126). The balance between commensalism and infection should be considered from the perspectives of both the host and the microbe, with oxygen availability a key factor. Studies focused on the gastrointestinal environment are beginning to reveal answers. A screen for overexpression mutants that resulted in improved gastrointestinal colonization identified the transcription factor Crz2 as playing a role in the initial steps of colonization (130). Further investigation found that *CRZ2* was a hypoxic-responsive gene that was important for overcoming the acidic pH and bile encountered in the upper gastrointestinal tract (130).

The maintenance of a commensal lifestyle requires that *Candida* have access to appropriate nutrient sources. It is well known that nutrient limitation and several stressors including oxygen deprivation are inducers of filamentation (19). As a commensal in the intestine, *C. albicans* must be able to adapt to changing nutrient sources in this low-oxygen niche. Under low-oxygen conditions, *C. albicans* undergoes highly dynamic metabolic reprogramming that is essential for its survival in this environment (16, 50). A similar metabolic reprogramming during hypoxia has been observed for *C. neoformans* and is coregulated by the two transcription factors Pas2 and Rds2 (129). Research exploring the genetic circuitry that allows for carbon source switching in low-oxygen conditions revealed a hypoxia-specific circuitry in *C. albicans* regulated by the SWI/SNF chromatin remodeling complex (16). Null mutants for the complex subunit Snf5 showed reduced growth on nonpreferred carbon sources specifically under low-oxygen conditions and were unable to form hyphae in hypha-inducing conditions (16). *snf5* null mutants were unable to establish in the murine gut, indicating the need for carbon source flexibility in this low-oxygen environment. Importantly, *snf5* null mutants were unable to establish infection in a *Galleria* larva model and unable to damage murine macrophages and human enterocytes (16). Additionally, transcriptional control of intestinal colonization is regulated by the SREBP Cph2, which regulates expression of some low-oxygen-responsive genes involved in the TCA (tricarboxylic acid) cycle and glycolysis (78). The molecular mechanisms of hypoxia response metabolic switches and programming remain to be fully explored in other human pathogenic fungi, but this is an important research direction given the implications for virulence and antifungal drug susceptibility.

As discussed, regarding biofilm development, resident *C. albicans* cells of various host foci are likely to be surrounded by other microbes. To be a successful commensal, *C. albicans* must adapt to life among these coresidents. The bacterium *Staphylococcus epidermidis* has been shown to coinhabit *C. albicans* colonies even after prolonged periods of growth in hypoxic conditions. The relationship between these two organisms was highly synergistic, making it difficult to separate them in the lab (15). A retrospective analysis of genome sequences of *C. albicans* isolates from candidemia patients showed that *S. epidermidis* was often co-isolated and found in genome sequencing reads at significant abundances (14, 15). Additionally, *C. albicans* can transcriptionally adapt to grow as a polymicrobial biofilm with *P. aeruginosa* (48). These examples demonstrate that the ability to coinhabit a niche is critical for the survival of *C. albicans* as a commensal and likely contributes to its pathogenesis. Given the diverse microbiome of the host, more research is needed into the biology of commensal fungi in the context of being embedded in the host microbiome. Given the niches *Candida* spp. inhabit, hypoxic adaptation is likely to be tightly linked to these cross-kingdom relationships in the human microbiome.

In addition to self-regulation and microbiome-mediated regulation of *C. albicans* commensalism, the host plays an important role in regulating the balance between commensalism and pathogenesis in hypoxic niches in the body. Recent studies have specifically linked regulation and monitoring of *Candida* species growth and morphology through host HIF-1α activity. In the oral cavity, epithelial cells monitor the levels of *Candida* species through miRNA regulation (65). Oral epithelial cells can distinguish between low and high levels of *C. parapsilosis* and *C. albicans* through miRNA regulation driven by HIF-1α (65). In two recent studies, two natural products used in traditional medicine were shown to enhance antifungal activity through modulation of HIF-1α and IL-17 signaling in oropharyngeal candidiasis caused by *C. albicans* and *C. glabrata* (20, 99).

Connecting the microbiome with host-relevant signaling, it has been observed that the resident intestinal bacteria of the phylum *Bacteroidetes* mediated activation of HIF-1α, leading to the activation of innate immune effectors and production of the antimicrobial peptide LL-37 (45). This signaling between a resident intestinal microbe and the host results in regulation of *C. albicans* colonization in the intestine (45). This highlights the importance of the resident microbiome for maintaining an environment, in this case hypoxic conditions, that leads to a signaling cascade tipping the balance toward *C. albicans* commensalism, where a dysbiosis of the microbiome can tip the balance in the opposite direction, resulting in disease.

Outside of the gastrointestinal tract, hypoxic conditions that emerge during fungal infections may contribute to long-term host persistence phenotypes and have significant clinical implications. Interestingly, metabolic rewiring in *C. neoformans* can result in quiescence (64). Long-term exposure to low nutrients and hypoxic conditions leads to the formation of a subpopulation of “viable but non-culturable” (VBNC) cells that are metabolically quiescent and capable of enduring stressful growth environments (64). Importantly, these quiescent cells can be reactivated through exposure to pantothenic acid, a molecule involved in quorum sensing (64). VBNC cells are an interesting and important phenomenon that warrants exploration in other pathogenic fungi.

Along these lines, culturing of *A. fumigatus* from patient samples often fails to produce viable colonies. One possibility is that *A. fumigatus* enters a VBNC-like state in host tissues. Cystic fibrosis (CF) is characterized by a mutation in the transmembrane conductance regulator protein (CFTR) that results in a thick mucous layer and impairment of immune function (31). *A. fumigatus* is one of the most common fungal pathogens associated with CF and is found in the lungs of 60% of people with CF and is associated with faster decline in lung function (40). For *A. fumigatus* to persist in the CF lung, it must be able to adapt to changing oxygen gradients, oxidative stress, and osmotic stress resulting from cellular metabolism and host-microbe interactions in CF lungs. To better understand how *A. fumigatus* adapts to the CF lung environment, Ross et al. (107) analyzed a longitudinal series of 29 *A. fumigatus* isolates collected from a single patient with CF for 4.5 years. The study identified a single-nucleotide polymorphism within the gene encoding a mitogen-activated protein kinase kinase (*pbs2*) in one of the two persistent lineage isolates that likely evolved in the lungs of this CF patient. *Pbs2* encodes a high-osmolarity glycerol (HOG) pathway component that regulates the fungal response to hypoxic, oxidative, and osmotic stresses (87). These persistent *A. fumigatus* isolates exhibited reduced fitness in standard laboratory culture conditions (normoxic) with increased resistance to osmotic stress. These isolates, however, were able to grow to levels similar to that of the wild-type laboratory strain in low-oxygen conditions. Interestingly, the persistent isolates were also resistant to voriconazole despite the patient not previously having been treated with azole antifungals (107). How fungal persistence in patients with no azole exposure drives antifungal resistance is yet to be defined. Still, these studies highlight that a low-oxygen environment promotes in vivo fungal host adaptation, with implications for susceptibility to contemporary antifungal agents. How these strains evolve in vivo to adapt to the low-oxygen CF lung environment is an intriguing question. A study by Engel et al. (41) identified diploid *A. fumigatus* isolates from six people with CF that suggest parasexual recombination in vivo could be one mechanism. *Candida* spp. are also frequently isolated from people with CF, and in vivo host evolution of *C. lusitaniae* isolates in this context (people with CF) also results in reduced antifungal susceptibility in the absence of known antifungal drug exposure (32, 33). The potential for conserved hypoxia response–mediated metabolic rewiring driving reduced susceptibility to antifungal drugs in vivo is worth further investigation.

## CONCLUDING THOUGHTS AND FUTURE DIRECTIONS

Oxygen is central to the physiology of human fungal pathogens and the host cells they interact with to mediate disease outcomes. As oxygen is limiting, or becomes limiting, at sites of infection initiation and progression, and in fungal biofilms themselves, the fungal hypoxia response is central to infection outcomes in the context of contemporary antifungal therapies. Thus, there is a strong argument for targeting the fungal hypoxia response to improve infection outcomes, and continued research on the underlying mechanisms is needed. A strong research theme in recent years is the metabolic rewiring of fungal and host cells in response to oxygen deprivation that alters the host-fungal interaction and contributes to reduced antifungal drug susceptibility (Figure 1). A fundamental gap in knowledge in this regard is the key factors that sense oxygen in fungal cells and activate the subsequent downstream signaling pathways. Defining these factors and mechanisms may yield new therapeutic opportunities. For example, the PHD oxygen sensors in mammalian cells are druggable targets whose activation or inhibition can dramatically impact cellular physiology. Also, targeting key fungal hypoxia response regulatory pathways involved in regulated proteolysis mediated by ubiquitin ligases and proteases may be promising. Finally, understanding the intricate metabolic rewiring that occurs when oxygen is limiting to the respective fungal pathogens is expected to reveal new infection-site-specific therapeutic targets. Here, the concept of VBNC cells in more chronic fungal infections, such as those found in people with CF, warrants further study. In this regard, it will be important moving forward to conduct high-throughput chemical and stress-related screens in fungi under conditions of in vivo– relevant oxygen levels to identify new antifungal chemical matter and potentially new infection site–relevant drug targets.

## Figures and Tables

**Table 1:** Genes associated with the fungal hypoxia response

Gene	Protein function
*ACE2*	Transcription factor that promotes filamentous growth in hypoxia in *Candida albicans*
*agsA*	1,3-α-Glucan synthase upregulated in hypoxia in *Aspergillus fumigatus*; may play a role in hypoxia fitness and virulence
*AHR1*	Negative regulator of hyphal growth in hypoxia in *Candida albicans* via Efg1-mediated pathway
*aoxA*	Alternative oxidase that promotes resistance to oxidative stress and increases virulence in *Aspergillus fumigatus*
*atrR*	Transcriptional regulator of genes involved in hypoxia adaptation and azole resistance in *Aspergillus fumigatus*; null mutant shows attenuated virulence
*bafA*	Subtelomeric cryptic gene; overexpression promotes hypoxic growth and hypoxia-locked phenotype in *Aspergillus fumigatus*
*bycA*	Amino acid permease that suppresses hyphal growth; confers calcineurin inhibitor resistance or loss of CnbR in *Mucor lusitanicus*
*catA*	Conidial catalase (ROS-scavenging gene) regulated by MybA and important for conidial survival in *Aspergillus fumigatus*
*CCR4-POP2*	An mRNA deadenylase required for cell wall biogenesis in *Candida albicans*; mutants show reduced cell wall β-glucans and reduced virulence
*chsE*	Chitin synthase upregulated in hypoxia in *Aspergillus fumigatus*; may play a role in hypoxia fitness and virulence
*chsF*	Chitin synthase upregulated in hypoxia in *Aspergillus fumigatus*; may play a role in hypoxia fitness and virulence
*cnbR*	Calcineurin regulatory B subunit that regulates the dimorphic transition from yeast to hyphae and is linked to virulence in *Mucor lusitanicus*
*coa6*	Mitochondrial respiratory complex IV assembly protein required for hypoxic growth in *Aspergillus fumigatus*
*CPH2*	Sterol regulatory element–binding protein that regulates hypoxia-responsive genes in glycolysis and the TCA cycle in *Candida albicans*; important for colonization of host gastrointestinal tract
*creA*	Transcriptional repressor important for growth in low oxygen in *Aspergillus fumigatus*; null mutant shows attenuated virulence
*CRZ2*	Hypoxia-responsive gene linked to cell wall function in *Candida albicans*; helps overcome acidic pH and bile in the host upper gastrointestinal tract
*crzA*	Calcineurin effector protein crucial for polarized hyphal growth and virulence in *Aspergillus fumigatus*
*cyp51a*	Ergosterol biosynthesis gene and target of AtrR and SrbA in *Aspergillus fumigatus*
*CYR1*	Adenylate cyclase; interacts with Snf5 to regulate carbon metabolism in hypoxia in *Candida albicans*
*ECE1*	Extent of cell elongation 1; hyphal-associated gene associated with hyphal elongation, adhesion, and biofilm formation in *Candida albicans*
*EFG1*	Transcription factor involved in filamentous growth of *Candida albicans* in normoxia; represses filamentous growth in hypoxia
*erg25a*	C4-sterol methyl oxidase regulated by SrbA; involved in hypoxia adaptation but not required for virulence in *Aspergillus fumigatus*
*erg3b*	Ergosterol biosynthesis gene and target of AtrR and SrbA in *Aspergillus fumigatus*
*FGF2*	Mammalian proangiogenic growth factor and a signaling protein
*fglA*	Heme-binding fungoglobin that is a potential oxygen sensor in *Aspergillus fumigatus*; required for optimal growth when oxygen level is critically low
*fksA*	1,3-β-Glucan synthase upregulated in hypoxia in *Aspergillus fumigatus*; may play a role in hypoxia fitness and virulence
*GOA1*	Mitochondrial protein in *Candida albicans*; null mutant shows increased oxidative stress sensitivity, reduced mitochondrial membrane potential, and inhibited hypoxia-induced β-glucan masking
*gpb1*	G-protein beta subunit that promotes hyphal growth in hypoxia and promotes virulence in *Mucor circinelloides*
*HDA1*	Histone deacetylase that regulates Ume6 in *Candida albicans*
*HIF-1α*	Hypoxia-inducible factor 1α; regulates mammalian gene expression in hypoxia
*hrmA*	Transcriptional regulator of a subtelomeric gene cluster; involved in hypoxia adaptation and development of hypoxia-locked morphology in *Aspergillus fumigatus*
*IFU5*	WW domain–containing protein that associates with Efg1 in normoxia and mediates adaptation to hypoxia in *Candida albicans*
*MAR1*	Cell surface–remodeling protein that regulates cell wall composition and immune evasion and is resistant to hypoxic stress in *Cryptococcus neoformans*; null mutant is hypovirulent
*MDR1*	Multidrug resistance transporter in *Candida albicans* that mediates azole resistance
*mybA*	Transcription factor required for conidiogenesis and conidia viability and is important for virulence in *Aspergillus fumigatus*
*OFD1*	Prolyl hydroxylase that degrades Sre1 in the presence of oxygen and acts as an indirect sensor of oxygen in fission yeast
*PAS2*	Transcription factor that forms a complex with Rds2 to mediate metabolic adaptation to hypoxia in *Cryptococcus neoformans*
*pbs2*	Mitogen-activated protein kinase kinase; regulates adaptive growth response to hypoxic and osmotic stress in *Aspergillus fumigatus*
*pkr1*	Protein kinase A regulatory subunit that promotes hyphal growth in hypoxia and is involved in the transition from anaerobic yeast growth to aerobic hyphal growth in *Mucor circinelloides*
*racA*	Small GTPase required for proper hyphal growth and conidiogenesis with no effect on virulence in *Aspergillus fumigatus*
*RAD53*	DNA checkpoint effector; reduces biofilm formation and increases fluconazole susceptibility in hypoxia in *Candida glabrata*
*RDS2*	Transcription factor that forms a complex with Pas2 to mediate metabolic adaptation to hypoxia in *Cryptococcus neoformans*
*SNF5*	Chromatin-remodeling complex subunit that activates carbon metabolism in hypoxia in *Candida albicans*
*srbA*	Sterol regulatory element–binding protein required for hypoxia adaptation and virulence in *Aspergillus fumigatus*
*srbB*	Sterol regulatory element–binding protein involved in heme biosynthesis and C4-sterol demethylation and a target of SrbA in *Aspergillus fumigatus*; important for hypoxia adaptation and virulence
*SRE1*	Sterol regulatory element–binding protein activated by both low oxygen and sterol depletion to activate genes required for hypoxia adaptation in *Schizosaccharomyces pombe*
*TYE7*	Transcriptional activator of glycolic genes and negative regulator of hyphal growth in hypoxia in *Candida albicans*
*uge3*	UDP-glucose-epimerase involved in galactosaminogalactan synthesis; null mutant shows decreased adherence to host cells and attenuated virulence in *Aspergillus fumigatus*
*UME6*	Hyphal-specific transcription factor involved in the initiation and maintenance of hyphal growth in hypoxia in *Candida albicans*
*UPC2*	Transcription factor that regulates sterol biosynthesis and azole drug resistance in *Candida albicans*
*WOR1*	Transcription factor that promotes white-opaque switching and enhanced fitness in *Candida albicans* in the host digestive tract
*XOG1*	Exoglucanase that promotes β-glucan masking in hypoxia; *Candida albicans* null mutants show attenuated virulence

Abbreviations: ROS, reactive oxygen species; TCA, tricarboxylic acid.

## Abbreviations

Normoxia: normal oxygen availability (atmospheric oxygen tension of approximately 21% O_2_ or pO_2_ of 160 mmHg at sea level) or oxygen availability sufficient to meet the physiological oxygen demands of the cell (2.5-9% in most human tissues)
Hypoxia: insufficient oxygen availability to maintain homeostasis or meet the physiological oxygen demands of the cell (in humans, typically oxygen levels of ≤5%)
Microoxia: an environment with only trace amounts of available oxygen, typically used in the context of controlled laboratory experimental conditions
Anoxic: describes complete lack of oxygen in an environment; a state of severe hypoxia in which aerobic processes cannot occur
Anaerobic: describes processes or environments using no or extremely limited amounts of molecular oxygen
